# A Review on Non-thermal Atmospheric Plasma for Food Preservation: Mode of Action, Determinants of Effectiveness, and Applications

**DOI:** 10.3389/fmicb.2019.00622

**Published:** 2019-04-02

**Authors:** Mercedes López, Tamara Calvo, Miguel Prieto, Rodolfo Múgica-Vidal, Ignacio Muro-Fraguas, Fernando Alba-Elías, Avelino Alvarez-Ordóñez

**Affiliations:** ^1^Department of Food Hygiene and Technology, Universidad de León, León, Spain; ^2^Institute of Food Science and Technology, Universidad de León, León, Spain; ^3^Department of Mechanical Engineering, Universidad de La Rioja, Logroño, Spain

**Keywords:** plasma, food safety, food quality, microbial inactivation, food processing

## Abstract

Non-thermal Atmospheric Plasma (NTAP) is a cutting-edge technology which has gained much attention during the last decade in the food-processing sector as a promising technology for food preservation and maintenance of food safety, with minimal impact on the quality attributes of foods, thanks to its effectiveness in microbial inactivation, including of pathogens, spoilage fungi and bacterial spores, simple design, ease of use, cost-effective operation, short treatment times, lack of toxic effects, and significant reduction of water consumption. This review article provides a general overview of the principles of operation and applications of NTAP in the agri-food sector. In particular, the numerous studies carried out in the last decade aimed at deciphering the influence of different environmental factors and processing parameters on the microbial inactivation attained are discussed. In addition, this review also considers some important studies aimed at elucidating the complex mechanism of microbial inactivation by NTAP. Finally, other potential applications of NTAP in the agri-food sector, apart from food decontamination, are briefly described, and some limitations for the immediate industrial implementation of NTAP are discussed (e.g., impact on the nutritional and sensory quality of treated foods; knowledge on the plasma components and reactive species responsible for the antimicrobial activity; possible toxicity of some of the chemical species generated; scale-up by designing fit-for-purpose equipment).

## Introduction

At present, consumers demand safe foods which also meet other quality criteria that traditional food preservation methods do not meet, such as convenience, high nutritional and sensorial quality, long shelf life, freshness, additives-free status, environment-friendly processing and low production costs (Fernández et al., 2012). Heat treatments have been widely used for many years to extend shelf life and enhance food safety and are generally well-accepted by consumers. However, they present certain drawbacks, such as the loss of nutrients and the considerable reduction in the organoleptic quality of some foods they cause (Jayasena et al., 2015). For this reason, a great deal of attention has been paid in recent years to the development of new preservation approaches, based on novel technologies, so-called “Emerging Food Preservation Technologies,” whose objective is to inactivate microorganisms and enzymes without appreciably altering their nutritional, organoleptic and functional characteristics (Kim et al., 2014). These novel technologies include high hydrostatic pressures, pulsed electric fields, high-intensity ultrasounds, pulsed light, oscillating magnetic fields and, more recently, non-thermal atmospheric plasma (NTAP).

The term Plasma is used in Physics and Chemistry to designate the state of an ionized gas. Plasma is considered the fourth state of Matter. Although solid, liquid and gaseous states are more common on Earth due to their temperature and pressure conditions, they are, in global terms, exceptional, whereas plasma is the predominant state across the universe. It is estimated that up to 99% of Matter is found in this state, e.g., in auroras, the ionosphere, the solar wind, the sun and other stars (Lackmann and Bandow, 2014). In addition, it is also possible to artificially produce plasmas, and many of them are part of our daily lives (e.g., televisions or monitors with plasma screens, fluorescent tubes or neon lamps used in lighting and as indicators in equipment, etc.). They are also used for industrial purposes, e.g., to confer certain functional properties onto materials (paper, plastics, fabrics or electronic elements), to produce electrostatic precipitation of fine particles, to generate ozone, or as an active medium for chemical synthesis processes.

In order to produce plasma it is necessary to supply energy to a gas to cause its ionization. Plasmas can be classified according to their temperature into two large groups: thermal plasmas and cold plasmas (Lieberman and Lichtenberg, 2005). Thermal plasmas can reach temperatures of up to several thousand Celsius degrees and are used in applications where high temperatures are required, such as in casting processes in the metallurgical industry or in chemical synthesis processes (e.g., production of acetylene from natural gas). Cold plasmas, with temperatures close to ambient temperature, are on the contrary suitable for the treatment of heat sensitive materials.

These cold or non-thermal plasmas (NTAP) are generated by the application of an electric or electromagnetic field to a gas. The field energy causes the free electrons to accelerate and ionizes the gas atoms and molecules, which release more free electrons that in turn provoke new ionizations. In addition, excited electrons produce molecular dissociations, with the formation of new atoms and free radicals, also able to excite atoms and molecules to higher energy levels. Excited atoms and molecules, when returning to the more stable state, emit excess energy in the form of broad-spectrum electromagnetic radiation, including ultraviolet (UV) radiation. Consequently, the plasma is constituted basically by molecules and atoms in an excited state, positive and negative ions, free radicals, electrons, UV radiation and reactive oxygen and nitrogen species, such as ozone, superoxide, hydroxyl radicals, singlet oxygen, atomic oxygen, nitric oxide or nitrogen dioxide. Interestingly, all these agents show antimicrobial activity against a wide range of microorganisms, including bacteria, molds, yeasts, and even bacterial and fungal spores (Klämpfl et al., 2012; Tseng et al., 2012; Takamatsu et al., 2015; Dasan et al., 2016).

## Potential of NTAP as a Preservation Technology in the Food Industry

The possibility of using plasma as a surface decontamination technology was first pointed out in the late 60's in an American patent (Menashi, 1968). However, NTAP was not implemented yet in the food industry at that point, since cold plasmas could only be obtained under vacuum and at small-scale conditions, which was expensive and not applicable in industrial settings (Lackmann and Bandow, 2014). Nonetheless, the technological advances experienced at the late 90's in relation to the sources of plasma generation allowed the development of equipment capable of generating plasmas at atmospheric pressure, thus avoiding the need for vacuum cameras and pumps and facilitating continuous treatments with relatively simple and inexpensive equipment.

NTAP offers very important advantages for food industries, which makes it a very promising novel food preservation technology. Firstly, it allows short processing times. Indeed, it has been described that very short treatment times (between a few seconds and 2 min) can cause more than 5 log reductions for different microorganisms, including pathogens such as *Salmonella* Typhimurium, *S*. Enteritidis, *Escherichia coli, Staphylococcus aureus, Listeria monocytogenes, Campylobacter jejuni, Campylobacter coli, Aeromonas hydrophila*, and even sporulated microorganisms such as *Bacillus cereus* and *Clostridium botulinum* (Deng et al., 2007; Muranyi et al., 2007; Rowan et al., 2007; Song et al., 2009; Shi et al., 2011; Lee et al., 2012b; Jahid et al., 2014; Ziuzina et al., 2014). In addition, it is effective at room temperature, which makes it particularly interesting for heat-sensitive products, and can be used to treat pre-packaged foods (Fröhling et al., 2012a; Rød et al., 2012; Ziuzina et al., 2014; Jayasena et al., 2015), which prevents their subsequent recontamination. Finally, its non-toxic nature and the reduced consumption of water and chemical agents result in a significant reduction of effluents, which is beneficial not only from an economic but also from an environmental point of view.

This set of advantages has led in recent years to explore the use of NTAP for food preservation, and there are already numerous studies, focused on characterizing its antimicrobial effectiveness and on deciphering the inactivation mechanisms involved. Nevertheless, a great research effort is still necessary to accomplish its successful implementation at industrial level as a safe and effective alternative to traditional preservation methods, with the main challenges arising from the difficulty in interpreting the data obtained by different research groups which use very diverse equipment and operating conditions, resulting in very different plasmas in terms of properties and, consequently, with very different antimicrobial effectiveness. However, some general conclusions can be drawn on various aspects related to the mechanisms of microbial inactivation by NTAP and the factors that determine its lethal efficacy, which will be discussed in the following sections of this review article.

## Mechanisms of Microbial Inactivation by NTAP

Although several studies have tried to elucidate the mode of microbial inactivation by various plasmas obtained under atmospheric conditions, the specific mechanisms leading to microbial death are not precisely known yet.

It is well-known that UV radiation with wavelengths in the 220–280 nm range is capable of inhibiting microbial growth by inducing the formation of DNA thymine dimers. Indeed, UV light has been used for years for the decontamination of water, air and surfaces. However, the contribution of UV radiation to the antimicrobial effect of plasmas obtained at atmospheric pressure is controversial. Thus, although some researchers hypothesize that UV-C radiation present in plasma plays an important inactivating role (Boudam et al., 2006; Eto et al., 2008; Muranyi et al., 2010), most authors (Laroussi and Leipold, 2004; Deng et al., 2006; Lee et al., 2006; Dobrynin et al., 2009, 2011; Joshi et al., 2011; Miao and Yun, 2011; Reineke et al., 2015) believe that UV radiations are not generated at the most effective wavelengths or are absorbed by the gas molecules themselves (Reineke et al., 2015) and, therefore, are not involved in microbial inactivation (Patil et al., 2014; Surowsky et al., 2014). Indeed, Reineke et al. (2015) compared the effectiveness of different plasmas for the inactivation of *Bacillus atrophaeus* and *Bacillus subtilis* spores and found that, although plasmas containing oxygen and nitrogen emitted four times more UV radiation than pure argon plasmas, the greatest lethal effect was achieved when pure argon was used as the working gas. These authors suggested that the antimicrobial effect was determined by reactive species of oxygen and nitrogen generated in the pure gas, and especially by hydroxyl radicals. Other authors have also tested the contribution of UV light by exposing *E. coli* cells (Liu et al., 2008; Gweon et al., 2009) and *Bacillus cereus* (van Bokhorst-van de Veen et al., 2015) and *B. subtilis* spores (Hong et al., 2009) to the action of plasma by intercalating between the plasma generation point and the treatment medium a lithium fluoride filter or a fused silica quartz plate transparent to UV light, which allows cell/spore exposure to the radiation potentially emitted by the plasma source but avoids contact with chemical reactive species. These studies demonstrated that the contribution of UV light to microbial inactivation was negligible, compared to that observed for direct exposition to plasma. In addition, exposure of *B. cereus* vegetative cells to nitrogen plasma has been shown to result in a transcriptional response in which the expression of genes involved in UV damage repair (*uvrA, uvrB*) was unaffected, while various genes involved in the response to oxidative stress, such as those encoding nitric oxide dioxygenase, as well as membrane-associated enzymes that catalyze oxidation-reduction reactions, were overexpressed (Mols et al., 2013). Finally, it has also been shown that microorganisms exhibiting variability in resistance to UV light exhibit a similar tolerance against NTAP. For instance, although *B. cereus* spores were more sensitive to UV light than spores of *Geobacillus stearothermophilus* and *B. atrophaeus*, a given NTAP treatment produced a similar degree of inactivation for the three species (van Bokhorst-van de Veen et al., 2015), suggesting that the mechanism of inactivation through both technologies was different.

There exists on the contrary a general agreement in that reactive chemical species generated through gas ionization exert an antimicrobial effect through a direct and non-specific attack on various microbial structures and components, including cellular envelopes, DNA and proteins (Song et al., 2009; Colagar et al., 2010; Surowsky et al., 2014; Yost and Joshi, 2015).

### Lesions at the Cellular Envelopes

Various authors (Dobrynin et al., 2009; Muranyi et al., 2010; Miao and Yun, 2011; Tseng et al., 2012; Tian et al., 2015) have identified the mechanical or oxidative damage caused to cellular envelopes as the main cause of death after NTAP treatments. The mechanical erosion of cellular envelopes could be due either to the impact of plasma energy particles, such as electrons and excited atoms (Butscher et al., 2016), or to the accumulation of charges in certain parts of the cell surface, which would lead to its permeabilization through the formation of pores, in a phenomenon known as electroporation (Laroussi et al., 2003). Moreover, damage at this level would facilitate the release of important intracellular components and the invasion of reactive species which would attack other cellular components, such as DNA and proteins, thus accelerating the inactivation process. In addition to this mechanical effect, neutral reactive species generated in plasma, such as atomic oxygen, hydroxyl and peroxyl radicals, metastable oxygen or ozone, are capable of causing oxidative damage to various cellular structures and macromolecules. If this damage is very intense and exceeds the physiological repair capacity, it will lead to microbial death (Leipold et al., 2010). In fact, the use of substances that chelate reactive species has been shown to reduce the antimicrobial efficacy of NTAP. Thus, several sequestering agents of various reactive oxygen species, such as catalase, superoxide dismutase, dimethyl sulfoxide, thiourea, sodium azide, and various antioxidants (glutathione, ascorbic acid, sodium pyruvate, mannitol, histidine, or tocopherol) have been reported to protect microorganisms, such as *E. coli* (Joshi et al., 2011; Yost and Joshi, 2015) or *P. aeruginosa* (Takamatsu et al., 2015), from the action of NTAP. Yost and Joshi (2015) showed that previous incubation of *E. coli* with catalase provided a greater protective effect than vitamin E and thiourea, concluding that peroxides such as H_2_O_2_ could be the major chemical species responsible for microbial inactivation by NTAP. On the other hand, Takamatsu et al. (2015) observed that the presence of dimethyl sulfoxide or sodium azide resulted in lower *P. aeruginosa* inactivation rates than those observed when catalase or superoxide dismutase were used, suggesting that hydroxyl radicals and singlet oxygen were the main agents involved in microbial inactivation.

Reactive species exert their oxidative effect especially on the polyunsaturated fatty acids of the cytoplasmic membrane (Laroussi and Leipold, 2004), which are very susceptible to lipid peroxidation phenomena. Lipid peroxidation can compromise cell viability by modifying membrane properties, decreasing its permeability and even its integrity (Joshi et al., 2011; Hosseinzadeh Colagar et al., 2013). Lipid peroxidation is a chain reaction that begins with the attack of unsaturated fatty acids by reactive oxygen species (or any reactive species), which extract a hydrogen atom from a methylene group (-CH2), giving rise to the formation of a lipid radical (L^*^) which can react rapidly with an oxygen molecule to give a peroxyl radical (LOO^*^). These radicals can extract new hydrogen atoms from other lipids and become hydroperoxides (LOOH), which undergo chemical degradative phenomena to produce very toxic degradation compounds, such as alkoxy (LO^*^), peroxyl and hydroxyl radicals and reactive aldehydes, including malondialdehyde and 4-hydroxynonenal. In fact, malondialdehyde is commonly used as a biological marker of oxidative stress (Liu et al., 2008), and a linear correlation between the content of this compound in *E. coli*, the time of exposure to NTAP and microbial viability has been described (Joshi et al., 2011; Hosseinzadeh Colagar et al., 2013; Yost and Joshi, 2015). These intermediate reactive compounds also behave as secondary toxic messengers of the reactive species generated in the plasma, amplifying their lethal damage to the cell (Joshi et al., 2011). Furthermore, many have greater stability than the plasma reactive species and, consequently, a much longer cytotoxic action, and can disseminate from the membrane toward more distant molecules, like DNA (Yost and Joshi, 2015), where they exert their action by interacting with nucleotides, inducing important modifications and cross-links and making cell growth and DNA repair more difficult (del Rio et al., 2005). In *S*. Typhimurium, malondialdehyde has been shown to be capable of inducing base insertions, deletions and substitutions, thus potentially contributing to significant DNA alterations (Dobrynin et al., 2009). In addition, some of the aldehydes generated in the peroxidation process, including malondialdehyde, are capable of forming cross-links in the polypeptide chains of proteins, affecting the activity of enzymes and membrane-associated proteins. To sum up, NTAP causes an uncontrolled process of lipid peroxidation which results in (i) changes in the membrane chemical composition, ultrastructural organization and permeability, (ii) a decrease in membrane fluidity, and (iii) the inactivation of membrane-associated enzymes, thereby compromising cellular viability.

The existence of damages in the cellular envelopes upon exposure to NTAP has been repeatedly demonstrated by using direct and indirect methods. Micrographs obtained through scanning electron microscopy for both vegetative cells (*Staphylococcus aureus, E. coli, B. subtilis, Campylobacter jejuni, Citrobacter freundii, Pseudomonas aeruginosa, Enterococcus faecalis, Saccharomyces cerevisiae, Candida albicans*) and bacterial (*B. cereus, B. subtilis* and *G. stearothermophilus*) and fungal (*Aspergillus parasiticus, Aspergillus flavus*) spores have allowed the identification of morphological alterations, such as size reductions, changes of shape, surface structure modifications, presence of pores in membranes and envelope disruptions (Yu et al., 2006; Hong et al., 2009; Miao and Jierong, 2009; Joshi et al., 2011; Miao and Yun, 2011; Bermúdez-Aguirre et al., 2013; Ryu et al., 2013; Zhang et al., 2013; Kim et al., 2014; Surowsky et al., 2014; Ma et al., 2015; van Bokhorst-van de Veen et al., 2015; Butscher et al., 2016; Cui et al., 2016a; Dasan et al., 2016; Gabriel et al., 2016b; Lai et al., 2016; Nishime et al., 2017). For bacterial spores, a loss of refringence, not accompanied by the onset of germination, has been observed for *B. cereus* by phase contrast microscopy (van Bokhorst-van de Veen et al., 2015). Damages in the cell envelopes have also been evidenced indirectly by determining the release of different intracellular components, including small ions such as K^+^ (Zhang et al., 2013), ATP (Cui et al., 2016a), nucleic acids (Zhang et al., 2013; Tian et al., 2015; Cui et al., 2016a), proteins (Liu et al., 2008; Miao and Jierong, 2009; Miao and Yun, 2011; Zhang et al., 2013; Tian et al., 2015; Cui et al., 2016a) or dipicolinic acid in the case of bacterial spores (Tseng et al., 2012), or by monitoring the entry into the cell of fluorescent dyes which cannot cross intact membranes, such as propidium iodide (Joshi et al., 2011; Surowsky et al., 2014; Ma et al., 2015; Tian et al., 2015), the decrease in membrane potential or its depolarization (Joshi et al., 2011; Surowsky et al., 2014; Tian et al., 2015; Yost and Joshi, 2015), as well as the inability of cells to retain the Gram staining (Muranyi et al., 2010; Joshi et al., 2011).

### Damages at Intracellular Level

However, it appears that microbial cell envelopes are not the only damaged cell structure. Indeed, reactive species, such as singlet oxygen, hydrogen peroxide, nitric oxide and excited atoms and molecules, can rapidly and easily diffuse into the cells, even when the membrane is intact, and oxidize many macromolecules (Dobrynin et al., 2011; Joshi et al., 2011; Yost and Joshi, 2015). Recently, Ziuzina et al. (2015b) detected and quantified, in *L. monocytogenes* and *E. coli*, the intracellular pool of reactive oxygen species after exposure to NTAP, using fluorescence techniques. They observed an increase in their concentration after NTAP treatment, which was more pronounced in *L. monocytogenes*. Similarly, Joshi et al. (2011), using highly selective fluorescent compounds for singlet oxygen and hydrogen peroxide quantification, found that the singlet oxygen concentration inside *E. coli* cells increased steadily within a 60 second NTAP treatment, whereas hydrogen peroxide content reached a maximum value after 12 s of treatment. In *S. cerevisiae*, an increase in the intracellular concentration of reactive species of oxygen and, especially, of nitric oxide has also been reported (Ryu et al., 2013). Once inside the cell, reactive substances provoke damage not only on lipids, but also on proteins, nucleic acids and carbohydrates. In order to neutralize the deleterious effects of such oxidant species or to repair the damage they cause, microorganisms have a number of mechanisms of elimination and transformation of these compounds, such as catalases, superoxide dismutases and glutathione and thioredoxin systems, among others. Nevertheless, when the accumulation of reactive oxygen species exceeds the cellular detoxifying capacity, and their concentration surpasses a critical level, the microbial cell suffers from oxidative stress, which induces the expression of certain genes and the activation of different defense responses (Pomposiello and Demple, 2002). For instance, in *E. coli*, the regulators *oxyRS* and *soxRS* are involved in the cellular response to oxidative stress induced by NTAP. Indeed, the exposure of *E. coli* to NTAP results in the overexpression of the *oxyS, soxS*, and *soxR* genes, as well as of those genes encoding for the catalase (KatG) and superoxide dismutase (SodA) enzymes (Sharma et al., 2009; Yost and Joshi, 2015). In addition, *E. coli* mutants lacking the *oxyR* or *soxS* genes have been shown to be more susceptible than wild-type strains to a plasma treatment using as working gases helium or a mixture of helium and oxygen (Perni et al., 2007). Similarly, Yost and Joshi (2015) reported that strains deficient in superoxide dismutase (SodA) were more rapidly inactivated by a NTAP treatment than wild-type strains. Overall, these results demonstrate the occurrence of reactive oxygen species within the cells during plasma treatments and suggest that the membrane is not the unique cellular component targeted by NTAP.

At the intracellular level, reactive species of oxygen and nitrogen, such as atomic oxygen, hydroxyl radicals (OH^*^), hydroperoxyls (HOO^*^), superoxides (O_2_-^*^) and nitric oxide (NO), are capable of interacting with various macromolecules and, according to some authors (Yost and Joshi, 2015), DNA and proteins are especially susceptible.

### DNA Damage

Damage to DNA is caused through the oxidation of bases. For example, oxidation of guanine generates 8-hydroxy-2'deoxyguanosine (OHdG), a compound which is used as a marker of oxidative DNA damage (Joshi et al., 2011; Yost and Joshi, 2015). The oxidation of DNA bases leads to a transversion (substitution of a purine for a pyrimidine or *vice versa*), which alters the bonds between DNA bases, causing changes in DNA conformation, and frequently leading to errors in the reading of the strand, mutagenesis and cell death. Some of the chemical species of plasma also react with deoxyribose carbons, breaking the N-glucosidic bond, thus generating apurinic or apyrimidine sites (abasic sites). All these changes may eventually lead to DNA strand ruptures (Hosseinzadeh Colagar et al., 2013; Ryu et al., 2013; Yost and Joshi, 2015). Microorganisms can nevertheless respond to DNA damage inflicted by NTAP. Indeed, transcriptional studies using microarrays have demonstrated that up to 18 genes involved in the SOS response are overexpressed upon exposure to NTAP (Sharma et al., 2009). Such overexpression is triggered when the level of accumulated damage is so high that microbial replication mechanisms are blocked. Similarly, Sharma et al. (2009), studying the transcriptomic response of *E. coli* cells exposed for 2 min to an argon plasma, observed an intense overexpression of several genes encoding for polymerase enzymes involved in the repair of extremely damaged DNA (*polB, dinB, umuC*, and *umuD*). In addition, these authors also detected an incomplete induction of some more specific DNA repair mechanisms, such as the nucleotide cleavage repair system, and concluded that plasma exerted massive damage at DNA level. The existence of significant damage to microbial DNA has also been corroborated using agarose gel electrophoresis techniques (Joshi et al., 2011; Han et al., 2013; Hosseinzadeh Colagar et al., 2013; Ryu et al., 2013). For instance, Joshi et al. (2011) observed fragmentation of *E. coli* DNA after NTAP treatment, which increased with increasing treatment times, and Hosseinzadeh Colagar et al. (2013) found that treatment times longer than 3 min resulted in a complete degradation of *E. coli* genomic DNA. Similar results have been obtained for *Saccharomyces cerevisiae* by Ryu et al. (2013), who also found complete DNA fragmentation after exposure to plasma for up to 3 min, and by Muranyi et al. (2010), who detected massive DNA damage in *B. subtilis* vegetative cells and, to a lesser extent, in their sporulated forms.

### Damage to Proteins

According to some authors, proteins and cytoplasmic enzymes would also suffer damage during plasma treatments. Indeed, reactive species are able to (i) break peptide bonds, (ii) oxidize amino acid side chains, especially of sulfur amino acids, such as methionine and cysteine, and amino acids harboring aromatic rings (tryptophan, phenylalanine and tyrosine), (iii) produce crosslinks within proteins, and (iv) give rise to aggregation phenomena, which are favored by the formation of intra- and inter-molecular disulfide bonds. All these effects result in modifications in the conformation and three-dimensional structure of proteins and enzymes (Surowsky et al., 2013). In addition, in the case of enzymes, their activity could also be compromised by the oxidation of their cofactors (Colagar et al., 2010). Actually, the oxidation of just one amino acid in a protein can affect its function (Lackmann et al., 2013). These latter authors showed that the loss of 3-phosphate glyceraldehyde activity after NTAP treatment was due to the oxidation of a cysteine residue (cys150) of its active site, which is known to be irreversible (Brandes et al., 2009). However, it is still unknown which enzymes and proteins are specific targets for the action of NTAP. In the particular case of vegetative cells, Hosseinzadeh Colagar et al. (2013) analyzed *E. coli* proteins after exposure to plasma using sodium dodecyl sulfate polyacrylamide gel electrophoresis (SDS-PAGE) techniques and found that high molecular weight proteins, of between 50 and 90 KDa, were especially sensitive to NTAP, observing a decrease in their content as treatment time increased. In addition, these authors also observed that the concentration of free amino acids linearly increased with exposure time, which suggested that NTAP is not only able to break peptide bonds, but also to fragment proteins liberating free amino acids. Concerning bacterial spores, several authors (Klämpfl et al., 2012; Tseng et al., 2012) have pointed out that the effectiveness of NTAP against these resistant forms is due to the inactivation of various enzymes responsible for spore germination, such as GerA and GerB, as well as to the inactivation of channel proteins involved in the transport of ions and dipicolinic acid, which also results in the inability to initiate germination processes.

### Indirect Mechanism of Action

Finally, apart from the direct effects caused by reactive species present in plasmas, an indirect mechanism of action, through the formation of other cytotoxic compounds, has been also described. Thus, Yost and Joshi (2015) showed that hydroxyl radicals (OH^*^) can be generated from H_2_O_2_ and superoxide radicals (${\text{O}}_{2-}^{*}$) in the presence of transition metals such as iron or copper. Although OH^*^ radicals can be produced by different routes, the Haber-Weiss reaction is the most habitual (Lemire et al., 2013). This reaction consists in the transformation of the ferric cation to ferrous cation (Fe^3+^ + ${\text{O}}_{2}^{{-}^{*}}$ → Fe^2+^ + O_2_) by the superoxide radical. Afterwards, the ferrous cation reacts with H_2_O_2_ to produce Fe^3+^, hydroxyl anions and hydroxyl radicals, which also exert and antimicrobial activity_._ Nitric oxide (NO) can also react with oxygen or superoxide radicals to form nitrogen dioxide (NO_2_), peroxynitrites (ONOO^−^ or ${\text{ONO}}_{2}^{-}$) and nitrous anhydride (N_2_O_3_) (Han et al., 2013), which all have a broad spectrum of antimicrobial activity, although they differ in reactivity, stability and biological activity (Fang, 2004).

## Factors Determining the Antimicrobial Effectiveness of NTAP

There are several factors known to influence the microbial inactivation achieved through NTAP treatments. Some of these factors are related to the conditions used for plasma generation and application, while others are associated with the particular properties of the microorganism targeted or with the characteristics of the treatment medium or food. The following sections of this review article will compile and discuss the information available in the literature in relation to these aspects.

### Processing Parameters

NTAP may be obtained through electrical discharges, which may be accomplished by using very diverse systems, e.g., corona discharge, micro hollow cathode discharge, atmospheric pressure plasma jet, gliding arc discharge, dielectric barrier discharge (DBD), radiofrequency (rf) and microwaves. In any case, electrical discharges are initiated and sustained through electron collision processes under the action of specific electric or electromagnetic fields. Among all the existing plasma sources, DBD and plasma jets are the most widely explored configurations in food research, due to their easiness of construction and adoption. Indeed, there are some commercially available DBD and plasma jet configurations. Nevertheless, regardless of the equipment used, the molecular composition of plasma will depend on the energy supplied, which in turn is determined by voltage, power and excitation frequency, and even on the composition and flow rate of the working gas, with these parameters being very variable among different NTAP units, which makes it very difficult to carry out direct comparisons among research studies executed employing different equipment and NTAP processing conditions. However, it is possible to draw some general conclusions on how some processing parameters affect the level of microbial inactivation achieved through NTAP treatments.

With processing times described in the literature ranging from a few seconds up to several minutes, it is well-known that microbial inactivation increases with processing time, although not in all cases exponentially. Thus, a wide variety of inactivation kinetics are observed in the published data, where it is frequent to find both linear, concave-upward (tailing) and concave-downward (shoulders) kinetics (Lee et al., 2011; Calvo et al., 2016, 2017).

Voltage (difference of potential between two points per unit of charge, expressed in Volts), power (electrical energy consumed per unit of time, expressed in Watts) and frequency (number of cycles per second, expressed in Hertz) have a great effect on the antimicrobial effectiveness of NTAP, as they are known to determine the input energy and, consequently, the chemical reactive species generated and their concentration. It has been repeatedly demonstrated that an increase in voltage increases microbial inactivation of both vegetative cells and bacterial spores (Table 1). In fact, Liu et al. (2013) have found, after using different voltages to generate helium plasmas, that the content of reactive chemical species and, in particular, of ${\text{N}}_{2}^{+}$, OH, He and O, and the level of inactivation achieved for *S. aureus*, increased with the voltage applied. It has also been shown that, working under a constant voltage, an increase in excitation frequency or in power gives rise to greater lethal effects in a wide range of microbial species (Table 1). Taking these findings into account it would be advisable to use high voltages, power, and frequencies for the generation of plasmas. However, some technological constraints, including high costs, and potential negative impacts on food quality, due to an excessive temperature rise, represent a limit toward the use of extreme processing conditions in industrial practice (Kim et al., 2011; Alkawareek et al., 2012; Daeschlein et al., 2012; Butscher et al., 2016).

**Table 1:** Summary of major research articles assessing the influence of processing parameters on the performance of non-thermal atmospheric plasma as a food decontamination technique.

**References**	**Treatment regime**	**Foods/medium**	**Microorganism**	**Main findings**
Marsili et al. (2002)	23 kV; Pulse rate: 320 pps; N_2_, CO_2_, air (10 L/min); Up to 50 s; batch	0.1% peptone solution	*E. coli* NCTC 9001, *S. aureus* NCTC 4135, *S*. Enteritidis NCTC PT4, *B. cereus* NCTC KD4	Air gas facilitated a greater inactivation after a 50 s treatment. *B. cereus* was the most susceptible microorganism.
Deng et al. (2007)	16–25 kV; 1,000– 2,500 Hz; Air; Up to 30 s	Almonds	*E. coli* 12955 (Collection of the Dept of Food Science and Nutrition, University of Minnesota)	Effectiveness increased with the applied voltage and the frequency. Cells at log growth phase were more sensitive than those at stationary phase.
Rowan et al. (2007)	23.5 kV; Pulse rate: 124 pps; N_2_, O_2_, CO_2_, air (10 L/min); Up to 30 s; 4°C; batch	Distilled water	*B. cereus* NCTC 11145 spores	The order of effectiveness was: O_2_ >CO_2_; air >N_2_
Muranyi et al. (2008)	170 W; Air (0–80% RH); 20°C; For up to 7 s	PET foils	*Bacillus subtilis* DSM 4181 spores, *Aspergillus niger* DSM 1957 spores	For *A. niger*, increased effectiveness at relative gas humidity of 70%; in contrast, *B. subtilis* spores showed slightly poorer inactivation at high gas humidity.
Miao and Jierong (2009)	13.56 MHz; 20–120 W; O_2_ (20–100 cm^3^/min); Up to 120 s	Poly(vinyl chloride) (PVC)	E. coli^*^	The optimum conditions (highest efficiency) are 100 W and O_2_ 60 cm^3^/min.
Song et al. (2009)	13.56 MHz; 75–150 W; Helium (10 L/min); Up to 120 s	Sliced cheese and ham	3-strain cocktail of *L. monocytogenes* (ATCC 19114, 19115, and 19111)	Increased effectiveness at high input power. Higher D values were obtained in sliced ham.
Jung et al. (2010)	13.56 MHz; 75 and 100 W; Helium and argon (6 L/min); Up to 120 s	Slide glass	*Bacillus subtilis* Spores^*^	Helium plasma was much less efficient than argon plasma; Increased effectiveness at high input power
Leipold et al. (2010)	10.4 kV; 21.7 kHz; 1.8 W-0.36 kW; Air; Up to 340 s	Knife	*Listeria innocua* (DMRI 0011)	Increased effectiveness at high discharge power
Ragni et al. (2010)	12.7 kHz; 15 kV; Air (RH 35 and 65%); 25°C; Up to 90 min	Shell eggs	*S*. Enteritidis MB2509, *S*. Typhimurium T5	Increased effectiveness at high air moisture content; *S*. Enteritidis resulted to be more plasma sensitive
Yun et al. (2010)	13.56 MHz; 75–150 W; Helium (4 L/min); Up to 120 s	Disposable plastic trays, aluminum foil, and paper cups	3-strain cocktail of *L. monocytogenes* (ATCC 19114, 19115, and 19111)	Increased effectiveness at high input power. The lowest D values were obtained on disposable plastic trays.
Kim et al. (2011)	13.56 MHz; 75–125 W; Helium (10 L/min) and He+O_2_ (10 sccm); Up to 90 s	Sliced bacon	*L. monocytogenes* (KCTC 3596), *E. coli* (KCTC 1682), *S*. Typhimurium (KCTC 1925)	Increased effectiveness at high input power and with the addition of oxygen to the working gas.
Lee et al. (2011)	2 kV; 50 kHz; He, N_2_ (7 L/min), He+O_2_, N_2_+O_2_ (0.07 L/min); Up to 2 min	Agar plates, slices of cooked chicken breast and ham	*L. monocytogenes* KCTC 3596	N_2_ was more effective than He. The addition of O_2_ to both gases improved their effectiveness, being N_2_ + O_2_ the most effective mixture. The highest and the lowest levels of inactivation were obtained on agar plates and sliced chicken breast, respectively.
Alkawareek et al. (2012)	6 kV; 20–40 kHz; 0.5% O2+ 99.5% helium (2 L/min); Up to 240 s	Agar plates	*Pseudomonas aeruginosa* PA01 (ATCC BAA-47)	Increased effectiveness at high frequencies
Fröhling et al. (2012b)	27.12 MHz; 10–40 W; Argon (20 L/min); Up to 4 min	Gelrite®-a polysaccharide gel	*L. innocua* DSM 20649; *E. coli* DSM 1116	Increased effectiveness at high discharge power
Niemira (2012)	47 kHz; 549 W; Air and N_2_; Distance from the plasma jet: 2–6 cm; Up to 20 s	Almonds	*S*. Anatum F4317, *S*. Stanley H0558, *S*. Enteritidis PT30, *E. coli* O157:H7 (C9490, ATCC 35150, ATCC 43894)	Air was more effective The greatest reductions were observed for *E. coli* O157:H7 C9490 at a 6 cm distance
Bermúdez-Aguirre et al. (2013)	3.95–12.83 kV; 60 Hz; Argon (455.33 sccm); Up to 10 min	Lettuce, baby carrots, tomatoes	*E. coli* ATCC 11775	Effectiveness increased with voltage and decreased with increasing contamination level. Tomatoes, followed by lettuce, were easier to decontaminate than carrots.
Han et al. (2013)	56 and 70 kV; 50 Hz; Air, 90% N_2_ + 10% O_2_, 65% O_2_ + 30% CO_2_ + 5% N_2_; Direct and indirect exposure; Up to 120 s; batch	Phosphate Buffered Saline (PBS)	*E. coli* ATCC 25922, *E. coli* NCTC 12900, *L. monocytogenes* NCTC11994	Greater reduction of viability using higher voltage and with working gas mixtures with higher oxygen content. Indirect mode of exposure more effective than direct exposure. *L. monocytogenes* resulted to be more sensitive
Ziuzina et al. (2013)	40 kV; Air; Direct and indirect exposure; Up to 300 s; batch	Maximum recovery diluent (MRD) or PBS	*E. coli* ATCC 25922	Direct exposure resulted to be more effective than indirect exposure, especially at lower plasma treatment times.
Patil et al. (2014)	50 Hz; 70 kV; Air (3–70% RH), 90% N_2_ + 10% O_2_, 65% O_2_ + 30% CO_2_ + 5% N_2_; 20°C; Direct and indirect exposure; Up to 120 s	Sterile polystyrene Petri dish containing *B. atrophaeus* spore strips	*B.atrophaeus* standard spore strips (Sportrol®_Namsa®)	The gas mixture 65% O_2_ + 30% CO_2_ + 5% N_2_ was the most effective; Increased effectiveness at high air moisture content and after direct exposure to plasma
Edelblute et al. (2015)	24 kV; 500 Hz; Air (5, 10 L/min); Up to 3 min	Brain Heart Infusion (BHI) agar plates	*Ecoli* ATCC 25922 and *S. epidermidis* ATCC 12228.	Effectiveness decreased with the flow rate
Takamatsu et al. (2015)	16 kHz; 9 kV; 10 W; Ar, O_2_, N_2_, CO_2_, air (1 L/min); 20°C; Up to 120 s; batch	PBS	*S. aureus* ATCC 25923, *P. aeruginosa* ATCC 27853	The greatest antimicrobial effect was obtained with N_2_ and CO_2_
Butscher et al. (2016)	6–10 kV; 5–15 kHz; Argon; Up to 60 min	Wheat grains; Polypropylene granules	*Geobacillus stearothermophilus* (ATCC 7953) spores	Higher efficiency by applying faster pulse frequency or higher pulse voltage. Less decontamination effect on wheat grains than on polypropylene
Calvo et al. (2016)	1 kHz; 1 W; O_2_, N_2_ (5–15 L/min); Up to 4 min	Polycarbonate membrane filters	*L. monocytogenes* CECT 4301, *L. innocua* CECT 910	A higher sensitivity to plasma was observed when the treatment was performed using air; increases in flow rate from 5 to 10 L/min caused an acceleration of bacterial inactivation when air was used; gas flow rate hardly affected NTAP efficiency when nitrogen was used.
Cui et al. (2016a)	300–600 W; N_2_; Up to 3 min	Lettuce; Stainless steel coupons	Biofilms of *Escherichia coli* O157:H7 (CICC 21530).	Significant lower inactivation was observed at 300 W; The combination of plasma and clove oil exhibited a remarkable synergistic effect
Cui et al. (2016b)	300–600 W; N_2_ (100 sccm); Up to 3 min	Eggshell	*Salmonella* Enteritidis (CICC 21482); *Salmonella* Typhimurium (CICC 22956)	Significant lower inactivation was observed at 300 W; The combination of plasma and thyme oil exhibited a remarkable synergistic effect
Gabriel et al. (2016a)	2.45 GHz; 450 and 650 W; Air (5 L/min); Up to 25 min; batch	Young coconut liquid endosperm	Multi-strain cocktails of *E. coli* O157:H7 (7 strains), *S*. enterica (5 strains), *L. monocytogenes* (2 strains) and spoilage bacteria (*Klebsiella* spp., *Staphylococcus* spp., and *Kluyvera* spp.)	Significant lower inactivation was observed at 450W; *S. enterica* exhibited the greatest plasma resistance, *while L. monocytogenes* and *Staphylococcus* spp. exhibited the least.
Lai et al. (2016)	4 W; Air (2–7 m/s); 52–90% RH	Sterilized distilled water	*Micrococcus luteus* ATCC 4698, *Staphylococcus epidermidis* ATCC 12228, *E. coli* ATCC 10536, *Serratia marcescens* ATCC 6911, *Pseudomonas. alcaligenes* ATCC 14909.	The inactivation efficacy increased with flow rate and decreased with relative humidity. The inactivation efficacy at 90% R.H. dropped to 10% of the value measured at 55% R.H.
Calvo et al. (2017)	1 kHz; 1 W; O_2_, N_2_ (5–15 L/min); Up to 12 min	Polycarbonate membrane filters	*S*. Typhimurium CECT 443, *S*. Enteritidis CECT 4300	Microbial inactivation was higher when air was used and with increasing flow rates

* *Non-specified strain*.

*RH: relative humidity; pps: pulses per second; sccm: standard cubic centimeters per minute*.

Another variable to take into account when considering the antimicrobial effectiveness of NTAP treatments is the working gas used. A wide variety of gases have been used to generate plasma, with the most frequent ones being nitrogen, oxygen, carbon dioxide, argon, helium, air, or mixtures of some of these gases (Table 1). All of them give rise to plasmas capable of achieving certain level of microbial inactivation, and there is no general agreement on which one is the most effective gas. Lee et al. (2011) compared the effectiveness against *L. monocytogenes* of plasmas obtained with helium or nitrogen and observed a higher inactivation level for nitrogen-based plasmas. Other studies have described that air-based plasmas are most effective in inactivating *E. coli* O157:H7, *B. cereus, S. aureus, L. monocytogenes, L. innocua* and various serovars of *Salmonella enterica*, such as *S*. Anatum, *S*. Stanley, *S*. Typhimurium and *S*. Enteritidis, than plasmas generated with nitrogen (Marsili et al., 2002; Niemira, 2012; Calvo et al., 2016, 2017). On the contrary, Takamatsu et al. (2015) obtained a greater lethal effect against *S. aureus* and *P. aeruginosa* with nitrogen and CO_2_ based plasmas than with plasmas generated with air, O_2_ or argon, and Rowan et al. (2007) concluded that oxygen is the gas of choice, over CO_2_ or nitrogen, when generating plasmas for the inactivation of *E. coli, C. jejuni, C. coli, L. monocytogenes, S*. Typhimurium*, S*. Enteritidis and *B. cereus* spores. On the other hand, it seems to exist an agreement, although with some exceptions (Reineke et al., 2015), in that the addition of small amounts of oxygen to noble gases, such as helium (Gweon et al., 2009; Kim et al., 2011, 2013; Lee et al., 2011, 2012a; Galvin et al., 2013) and argon (Surowsky et al., 2014), or nitrogen (Lee et al., 2011, 2012b) improves the antimicrobial effectiveness of NTAP against vegetative cells and spore-forming bacteria (Table 1). This effect is mainly attributed to a higher formation of reactive oxygen species, such as hydroxyl and hydroperoxyl radicals, atomic oxygen, hydrogen peroxide, singlet oxygen and ozone, all of them with a high antibacterial activity.

Another processing parameter determining the antimicrobial effectiveness of NTAP is the gas moisture content. In fact, it has been occasionally observed (Dobrynin et al., 2011) that the use of completely dry gases is ineffective for *E. coli* inactivation, and there are several studies which show that an increase in the water content of the gas improves its effectiveness (Table 1). Thus, Ragni et al. (2010) reported that an increase in the air relative humidity from 35 to 65% increased the inactivation of *S*. Enteritidis and *S*. Typhimurium from 2.5 to 4.5 log cycles, and attributed this effect to a higher concentration of hydroxyl radicals in the plasma. Similar results were found by Patil et al. (2014) for *B. atrophaeus* spores. These authors used plasmas with different moisture content (3, 10, 30, 50, and 70%), and obtained a 5 to 6 log reduction at humidities of 3 and 10%, while a complete inactivation was observed at higher humidities. These authors related this higher antimicrobial activity to the increased generation of numerous reactive species, such as N_2_O_5_, H_2_O_2_, HNO_4_, or hydroxyl radicals, and, especially, to the decomposition of ozone in the presence of water, with the consequent formation of highly oxidizing species, such as hydroxyl and hydroperoxyl radicals, superoxide anion and H_2_O_2_. On the contrary, a recent study by Lai et al. (2016) has shown that when the relative air humidity increased from 52 to 81% or from 62 to 81%, the inactivation efficiency of NTAP against *E. coli* and *Staphylococcus epidermidis* decreased by 87 and 58%, respectively, which was linked to a decrease in the concentration of negative ions in plasma. These apparently contradictory results could be explained by the existence of an optimum moisture value for achieving a maximum antimicrobial activity. For instance, in the particular case of *Aspergillus niger*, a progressive increase in microbial inactivation was observed up to a moisture content of 70%, which was the optimum moisture content for NTAP generation (Muranyi et al., 2008).

Gas flow rate also seems to influence the effectiveness of NTAP treatments (Table 1). Lai et al. (2016) reported a steady increase in *E. coli, S. epidermidis* and *P. alcaligenes* inactivation when air flow rates increased from 2 to 7 meters/s, linked to a linear rise in the concentration of negative ions in the generated plasma. Results from our research group have demonstrated that the effect of gas flow rate on *L. monocytogenes* and *L. innocua* inactivation through NTAP depended on the type of gas used to generate plasma. Indeed, increases in flow rate from 5 to 10 L/min caused an acceleration of bacterial inactivation when air was used, while an additional increase of gas flow from 10 to 15 L/min had a minor impact on microbial inactivation. On the other hand, gas flow rate hardly affected NTAP treatment efficiency when nitrogen was used to generate plasma (Calvo et al., 2016). A similar behavior was also observed for *S*. Enteritidis and *S*. Typhimurium (Calvo et al., 2017). In contrast, Edelblute et al. (2015) found that an increase in air flow rate from 5 to 10 liters per minute was accompanied by a loss of efficacy of NTAP for the inactivation of *E. coli* and *S. epidermidis*, and attributed this effect to a reduction in ozone and NO_2_ concentration. Moreover, Miao and Jierong (2009), using oxygen as working gas, suggested the existence of an optimal flow rate for the inactivation of *E. coli*, reporting that, once this optimal flow rate is surpassed, the lethal effect achieved is reduced. According to these authors, at low flow rates, the number of reactive species, mainly constituted by oxygen radicals, is lower than at high flow rates, but they have a higher average energy and, therefore, the likelihood of each one colliding with microbial cells increases and, consequently, also their antimicrobial effectiveness. However, at higher flow rates the number of reactive species will be higher, but they will have a lower average energy, and their antimicrobial action would be comparatively lower.

NTAP effectiveness can also depend on whether treatments are carried out directly or indirectly. In direct treatments, the product is physically located in the field where plasma is generated, and, therefore, it is in intimate contact with all the photons and chemical species produced. In contrast, in indirect treatments plasma is produced at some distance from the product, and is usually displaced toward it, using a rapid flow of the feed gas. In principle, one would expect that indirect treatments would be less effective in microbial inactivation. Patil et al. (2014) demonstrated that the direct exposure of *B. atrophaeus* spores to plasmas generated with gases of different composition (air; a mixture of nitrogen [90%] and oxygen [10%]; a mixture of oxygen [65%], CO_2_ [30%], and nitrogen [5%]) caused the inactivation of at least 6 log cycles, whereas for indirect exposures inactivation rates ranged from 2.1 to 6.3 log units, depending on the type of gas used. Nevertheless, other authors have obtained conflicting results. Thus, Han et al. (2013) found that indirect treatments were more effective against *E. coli* and *L. monocytogenes* than direct treatments, and suggested that this fact could be due to the recombination of reactive radicals with a short life before reaching the microorganisms in indirect treatments, giving rise to new chemical species with strong bactericidal effects. However, there are also some authors (Ziuzina et al., 2015a) who did not detect differences in effectiveness between direct and indirect treatments when treating *L. monocytogenes, E. coli* and *S. aureus* biofilms.

Finally, it should be noted that, in the case of indirect treatments, another parameter that seems to determine NTAP lethality is the distance between the point of plasma generation and the sample. Thus, the results obtained by several authors show that, in general, the antimicrobial effectiveness decreases as that distance increases (Gabriel et al., 2016b; Nishime et al., 2017).

### Microbial-Related Factors

One of the key advantages of NTAP in comparison to other non-thermal food preservation technologies is its ability to inactivate not only bacteria, molds and yeasts, but also mold ascospores and bacterial spores (Muranyi et al., 2007; Rowan et al., 2007; Shi et al., 2011; Klämpfl et al., 2012; Dasan et al., 2016).

In general, molds and yeasts are more tolerant against the action of plasma than bacteria, and vegetative cells are more sensitive than bacterial spores (Lee et al., 2006; Muranyi et al., 2007; Rowan et al., 2007; Hong et al., 2009; Shi et al., 2011; Klämpfl et al., 2012; Tseng et al., 2012; Takamatsu et al., 2015). However, results obtained by numerous authors studying the effectiveness of NTAP under the same experimental conditions for a wide range of microbial groups have demonstrated that differences in NTAP resistance among these microbial groups are not as marked as those observed for thermal and other non-thermal processing technologies.

Some studies, in particular, provide interesting insights into the inter-kingdom and inter-species variability in NTAP resistance. Klämpfl et al. (2012) evaluated the effectiveness of an air plasma for the inactivation of vegetative cells of 15 bacterial species, including *E. coli. P. aeruginosa, S. aureus, E. faecalis, B. cereus, B. pumilus*, and *C. difficile*, one yeast species (*Candida albicans*), and spores of four bacterial species (*B. subtilis, B. pumilus, B. atrophaeus* and *G. stearothermophilus*). They observed that after 30 s of NTAP treatment, between 4 and 6 log reductions were achieved for vegetative cells, while after a 1 min exposure under the same working conditions, from 1 (*G. stearothermophilus*) to 4 (*B. subtilis*) log reductions were attained for bacterial spores. Lee et al. (2006), using a plasma obtained from a mixture of helium and oxygen, also reported a higher sensitivity to NTAP for vegetative cells than for yeasts and bacterial spores, with D values of 0.3 min for *E. coli* and *S. aureus*, 2 min for *S. cerevisae*, and 14 min for *B subtilis* spores. Similarly, Takamatsu et al. (2015) found that treatments of around 1 min with a nitrogen-based plasma were required in order to achieve 6 log cycles of inactivation for *E. coli, P. aeruginosa, E. faecalis* and *S. aureus*, while 5 and 15 min were required to achieve the same inactivation levels for *Aspergillus niger* and *B. cereus* spores, respectively. Moreover, Tseng et al. (2012) described D values of 0.50 min for vegetative cells of *E. coli* and *B. subtilis*, while D values obtained for bacterial spores of species belonging to the genus *Bacillus, Geobacillus* and *Clostridium* (*B. subtilis, G. stearothermophilus, C. sporogenes, C. perfringens, C. difficile*, and *C. botulinum* Type A and type B) ranged between 2.66 (*C. perfringens*) and 8.04 (*C. botulinum* type A) min. Interestingly, spores of *G. stearothermophilus*, generally used as biological indicators of heat sterilization treatments given their extreme thermoresistance, showed a similar NTAP resistance to that of spores of *B. subtilis*, a mesophilic microorganism, suggesting that mechanisms of inactivation by heat and plasma differ, at least to a certain extent. Similar observations were also made by van Bokhorst-van de Veen et al. (2015), who hardly found differences in NTAP resistance among spores of *B. cereus, G. stearothermophilus* and *B. atrophaeus*, obtaining between 3.7 and 4.9 log reductions after a 20 min treatment with a nitrogen-based plasma, while identified large inter-species variations in spore resistance to heat, UV light and chemical oxidants (hydrogen peroxide and sodium hypochlorite).

The higher NTAP resistance exhibited by bacterial spores may be due to the low water content and the high concentration of dipicolinic acid in the spore core, the low permeability of the spore inner membrane, the very robust spore coat, which represents a physical barrier to external agents, the special conformation of the spore DNA, which is saturated by a group of soluble acid proteins (SASP), the presence of detoxifying enzymes in the spore cortex, or the absence of metabolic activity within the spore, which limits the activity of plasma-generated components (Henriques and Moran, 2007). In a recent study by Reineke et al. (2015) analyzing two *B. subtilis* mutant strains, unable to synthesize dipicolinic acid during sporulation and two of the major SASPs, respectively, it was reported that only this latter strain was more sensitive to NTAP than the wild type strain, what suggested that DNA stabilization through SASPs may be responsible, at least in part, for the increased resistance of bacterial spores against NTAP.

The higher NTAP resistance exhibited by yeasts and molds in comparison to vegetative bacterial cells could be due to differences between prokaryotic and eukaryotic cells in cellular structure and molecular composition. On the one hand, the fungal genome is protected by the nuclear membrane, which constitutes an additional diffusion barrier that would contribute to increase the cell resistance to DNA damaging agents. On the other hand, the fungal cell wall is very thick and consists of rigid layers of polysaccharides, which would provide further protection to the cell (Klämpfl et al., 2012).

In relation to bacterial vegetative cells, Gram-positive bacteria are generally considered to be more resistant to NTAP than Gram-negative bacteria due to the fact that they have a thicker layer of peptidoglycan in their cell wall, which increases the rigidity of cellular envelopes, making them more resistant to mechanical damage and hindering the diffusion of reactive species through the envelopes (Lee et al., 2006; Ziuzina et al., 2014, 2015a; Edelblute et al., 2015; Jayasena et al., 2015; Yong et al., 2015a; Puligundla et al., 2017). However, other studies have found similar resistance to NTAP for particular species of both bacterial groups (Lee et al., 2006; Klämpfl et al., 2012; Tseng et al., 2012; Takamatsu et al., 2015), and, even, some authors have described strains of *L. innocua* (Baier et al., 2014), *L. monocytogenes* (Gabriel et al., 2016a), *B. cereus* (Marsili et al., 2002), *S. aureus* and *E. faecalis* (Nishime et al., 2017) as being more sensitive to NTAP than strains of *E. coli, S*. Typhimurium, *S*. Enteritidis, *Vibrio parahaemolyticus* and *Pseudomonas aeruginosa*, respectively. These results seem to indicate that it is not possible to draw general conclusions on whether Gram-positive and Gram-negative bacteria have differential sensitiveness against NTAP.

Intraspecific variability in NTAP resistance may also exist. Indeed, Galvin et al. (2013) and Burts et al. (2009) have reported differences of around 1 log cycle of inactivation among *S. aureus* strains exposed either to helium-based or air-based plasmas. Similar results have been described by Niemira (2012) for a group of *E. coli* O157:H7 strains exposed to air plasmas.

It is well-known that the cellular physiological state markedly determines microbial resistance to different inactivation treatments. In fact, bacterial cells harvested during exponential growth phase are known to be more sensitive against heat, pulsed electric fields and high hydrostatic pressures than cells obtained in the stationary phase of growth (Alvarez et al., 2000; Martínez et al., 2003; Mañas and Mackey, 2004). However, based on the results available in the literature, the microbial physiological state exerts little or no influence at all on microbial resistance against NTAP. Thus, no significant differences in D-values were found among *E. coli* (Yu et al., 2006; Deng et al., 2007) and *S*. Typhimurium (Fernández et al., 2013) cultures obtained in logarithmic, early and late stationary phases of growth.

It is worth noting that NTAP treatments have been shown to be capable of inactivating different bacteria (e.g., *S. aureus, E. faecium, L. monocytogenes, E. coli* O157:H7, *A. hydrophila* or *P. aeruginosa*) in the form of biofilms (Jahid et al., 2014; Flynn et al., 2015; Ziuzina et al., 2015a,b). It is well-known that cells within biofilms exhibit increased resistance to various disinfectant agents commonly used in the food industry (Pan et al., 2006). Similarly, cells in planktonic state show a higher sensitiveness toward NTAP than cells within biofilms. For instance, Jahid et al. (2014) reported for *A. hydrophila* that a treatment with an oxygen plasma for 15 s caused a 7 log reduction in planktonic cells, whereas only 3 log reductions were obtained for cells forming a biofilm, even when the treatment time was prolonged for up to 5 min. The increased NTAP resistance of cells within biofilms has been attributed to the protective effect exerted by the biofilm's extracellular matrix, constituted mainly by polymeric substances, including polysaccharides, phospholipids, proteins, nucleic acids, and teicoic acids (Shi and Zhu, 2009), which represents a physical barrier hindering the penetration of the chemical reactive species occurring in plasma, which would then require longer treatment times to exert their antimicrobial action (Vleugels et al., 2005).

Microbial resistance to various inactivation treatments is largely determined by growth conditions, especially temperature and pH (Alvarez-Ordóñez et al., 2008; Álvarez-Ordóñez et al., 2009; Alvarez-Ordóñez et al., 2010). However, little attention has been paid so far to the impact of such environmental growth conditions on the effectiveness of NTAP treatments. Very few studies have assessed the effect of growth temperature on NTAP resistance. There exists some data for *S*. Typhimurium, *S*. Enteritidis, *L. monocytogenes* and *L. innocua*, grown in the range 10–45°C (Fernández et al., 2013; Calvo et al., 2016, 2017), and for *A. hydrophila*, grown at temperatures between 4 and 30°C (Jahid et al., 2014). From these studies it can be concluded that no significant differences in NTAP resistance occur at growth temperatures close to the optimum growth temperature (20 to 45°C), while at temperatures below 20°C a minor sensitization of cells to plasma occurred. On the other hand, in the particular case of *A. hydrophila* cells forming biofilms, resistance to NTAP exponentially increased with increasing growth temperatures, with times required to reduce the population in 5 log cycles being 1.84 min at 4°C and 25.33 min at 30°C (Jahid et al., 2014).

In relation to the influence of growth pH, our research team (Calvo et al., 2016, 2017) has recently demonstrated that the growth of *S*. Typhimurium, *S*. Enteritidis and *L. monocytogenes* in media acidified with different acids (acetic, ascorbic, citric, lactic, malic, and hydrochloric) at low pH conditions (pH 6.5, 5.4 or 4.5) did not significantly influence the antimicrobial effectiveness of NTAP treatments. The short-term exposure of these microorganisms to acid, cold or heat stress shocks prior to NTAP treatments did no modify their resistance either. Since cross-resistance adaptive responses did not take place, these findings allowed us to conclude that NTAP may be a first-choice technology to be included into food processing schemes following a hurdles technology approach in combination with acidification, mild heating or refrigeration.

Several authors have shown that the effectiveness of NTAP treatments decreases when dealing with high microbial loads. This fact has been shown in various substrates and for different microorganisms, including *E. coli* (Yu et al., 2006; Burts et al., 2009; Miao and Yun, 2011; Bermúdez-Aguirre et al., 2013), *S. aureus* (Burts et al., 2009), *S*. Typhimurium (Fernández et al., 2012), and *S. cerevisae* (Lee et al., 2006). However, in the case of bacterial spores the results are not so clear. Indeed, while some authors did not find differences in NTAP effectiveness depending on the initial spore concentration for different species of the genus *Bacillus* (Purevdorj et al., 2001; Lee et al., 2006), in a recent study, Butscher et al. (2016) observed that when the initial load of *G. stearothermophilus* spores in wheat grains was reduced from 10^7^ to 10^6^ cfu/g the degree of inactivation achieved through a 10 min NTAP treatment increased from 1.02 to 1.82 log units.

Several authors have attempted to explain the loss of antimicrobial efficacy of NTAP on substrates with high microbial loads. Fernández et al. (2012) suggested that with high microbial loads the abundance of reactive species per cell would not be sufficient to cause their death. Even, these reactive species would react with already inactivated microorganisms or with their cellular components (e.g., proteins), which could act as sequestering agents (Kamgang-Youbi et al., 2008), thus protecting the remaining living cells. In this regard, Fernández et al. (2012) found that the addition of heat-inactivated cells significantly reduced NTAP effectiveness against *S*. Typhimurium. In addition, when high microbial loads are present, microorganisms are arranged in a stacked structure, in the form of multiple layers, with the upper microbial layers, even if already inactivated, constituting a physical barrier to the penetration of plasma, therefore protecting those microorganisms located in the inner layers. This distribution of microorganisms in layers has been observed through electron microscopy for *B. subtilis* spores (Deng et al., 2006), as well as through fluorescence microscopy for *S*. Typhimurium (Fernández et al., 2012). However, it should be taken into account that in all the studies mentioned above, contamination levels and microbial loads were much higher than those expected in naturally contaminated food.

### Characteristics of the Treatment Medium or Food

The most important factors related to the treatment medium properties determining microbial resistance to NTAP are the medium composition, in the case of liquids and liquid foods, and the topography and certain physical properties, in the case of solid foods.

For liquids, only those plasma-generated reactive species with a relatively long life, such as ozone, atomic oxygen, nitric oxide or hydrogen peroxide, would have the capacity to diffuse through the medium and interact with microbial cells (Ziuzina et al., 2013). However, NTAP has been shown to be very effective in liquid media (Montenegro et al., 2002; Shi et al., 2011; Ziuzina et al., 2013; Surowsky et al., 2014), which has been attributed to the generation of new secondary reactive species through the interaction of the chemical species of plasma with each other or with water or other molecules present in the liquid or gaseous phase of the medium. For example, the coexistence of ozone and hydrogen peroxide in water results in highly reactive species such as hydroperoxides and hydroxyl radicals. In addition, atomic oxygen, upon reaction with water, can generate hydrogen peroxide and singlet oxygen (Surowsky et al., 2014; Takamatsu et al., 2015), and the interaction of nitric oxide and superoxide produces peroxynitrite, a highly reactive compound that can readily diffuse through cell membranes (Ercan et al., 2016).

Several authors have reported that NTAP treatment of water and other aqueous solutions causes an increase in both their electrical conductivity and oxidation-reduction potential (Tian et al., 2015; Xu et al., 2016). In addition, several studies have detected through various analytical techniques the presence of reactive oxygen species and reactive nitrogen species in plasma treated water (Ryu et al., 2013; Surowsky et al., 2014; Ercan et al., 2016). However, apart from the presence of these highly reactive species, the rapid acidification that NTAP treatment causes in non-buffered solutions, due to the dissociation of water and the formation of nitric acid and nitrous acid if nitrogen is available, is also believed to be responsible for the inactivation capacity of NTAP in liquid media. Several authors (Rowan et al., 2007; Chen et al., 2009; Naïtali et al., 2010; Ryu et al., 2013; Ma et al., 2015) have shown that the pH of plasma treated water decreases gradually with treatment time up to values close to pH 3.0. This same behavior has been observed in non-buffered saline solutions (Oehmigen et al., 2010; Ryu et al., 2013; Ziuzina et al., 2013), which would justify the greater antimicrobial effectiveness of NTAP in these media as compared to that observed in media containing substances with buffering capacity (Chen et al., 2009; Naïtali et al., 2010; Ryu et al., 2013; Ziuzina et al., 2013). However, several authors have observed that the level of microbial inactivation attained in NTAP treated media (reaching ~pH 3.0) is much higher than that observed for media acidified at pH 3.0 with different acidulants (Chen et al., 2009; Liu et al., 2010; Naïtali et al., 2010; Ercan et al., 2016). In order to explain this behavior, several authors have speculated about the possibility that low pH values could contribute to the stabilization of some of the reactive species generated in plasmas (Yost and Joshi, 2015) or to the formation of new compounds with antimicrobial potential (Naïtali et al., 2010). A possible synergistic effect of low pH and NTAP-derived reactive species on microbial inactivation has been also proposed (Oehmigen et al., 2010; Sun et al., 2012).

The influence of treatment medium composition on plasma effectiveness in liquid media has been scarcely studied, but some reports have shown that the presence of some salts, such as carbonates, phosphates and sodium chloride (Chen et al., 2009; Ryu et al., 2013), reduce the efficacy of NTAP treatments, through reacting with the chemically active species or due to their buffering capacity. The presence of organic matter can also influence the effectiveness of NTAP. Indeed, Rowan et al. (2007) observed a greater inactivation of various enteropathogenic microorganisms in poultry washing water than in distilled water, which was attributed to the formation of nitric acid and carbonic acid from fat and proteins present in the medium. On the other hand, the inactivation of molds and yeasts in complex media has been shown to be much less effective than in a saline solution or water (Ryu et al., 2013).

Regarding microbial inactivation on solid media, NTAP is a very efficient technology for surface decontamination of abiotic surfaces, such as stainless steels, various packaging materials, paper, glass and plastics of diverse nature (Muranyi et al., 2007; Leipold et al., 2010; Miao and Yun, 2011; Klämpfl et al., 2012; Patil et al., 2014; Butscher et al., 2016; Gabriel et al., 2016b). In addition, it is also highly effective for surface decontamination of a wide variety of foods, such as fruits, vegetables, spices, nuts, cereal grains, seeds, seaweed, meat and meat products, eggs, egg products, cheese slices, and dried squid shreds (Table 2). However, microbial survival on different solids is conditioned by surface characteristics, which will determine its possible heating during NTAP treatment, the degree of microbial adhesion, as well as the level of formation and/or adsorption of active species. In particular, surface roughness, porosity and topography are of special relevance (Song et al., 2009; Noriega et al., 2011). A large number of studies have demonstrated a great variability in NTAP effectiveness against both vegetative cells and bacterial spores when identical treatments were applied onto different surfaces (Yun et al., 2010; Noriega et al., 2011; Yong et al., 2015a; Butscher et al., 2016). For example, Miao and Yun (2011) studied the inactivation of *E. coli* on PET (polyethylene terephthalate), PVC (polytetrafluoroethylene) and PTFE (polytetrafluoroethylene-teflon), and obtained the greatest lethal effect on PET, while the lowest inactivation was found on PTFE. These authors attributed the observed differences to differences in surface wettability of the materials, which was higher for PET, followed by PVC and, finally, PTFE. Fernández et al. (2013) obtained 2.7 log reductions in *S*. Typhimurium viability on polycarbonate filters after a 2 min NTAP treatment, while it took 15 min to reach 2.7; 1.8 and 0.9 log cycles of inactivation on lettuce, strawberries and cut potato, respectively. Similarly, Lee et al. (2011) and Butscher et al. (2016), comparing the effectiveness of NTAP for the inactivation of *L. monocytogenes* and *G. stearothermophilus* on abiotic surfaces and foods, observed that the highest antimicrobial activities were obtained on abiotic surfaces, such as agar plates or polypropylene grains. In addition, it has also been demonstrated that the effectiveness of NTAP as a food decontamination technique varies among different foods. Thus, inactivation of *E. coli* was more effective on tomato than on lettuce (Bermúdez-Aguirre et al., 2013) or strawberries (Ziuzina et al., 2014), also being faster on carrots than on apples (Baier et al., 2015). For *L. monocytogenes* a greater lethal effect was also observed on cheese slices than on ham (Song et al., 2009), on sliced ham than on chicken breast filets (Lee et al., 2011), or on tomato than on strawberries (Ziuzina et al., 2014). These results show that food topography should be taken into account when designing effective NTAP treatments. Additionally, differences can be also attributed to the fact that bacteria could have been in the form of planktonic cultures on the surface, biofilms or internalized in the food tissues, and it is well-known that this can show a very important influence on the inactivation effectiveness of NTAP (Ziuzina et al., 2015b; Berardinelli et al., 2016).

**Table 2:** Summary of major research articles assessing the level of microbial inactivation achieved and the quality changes occurring in foods subjected to non-thermal atmospheric plasma treatments.

**Product**	**Treatment regime**	**Maximal log reduction**	**Impact on quality attributes**	**References**
**MEAT AND MEAT PRODUCTS**				
Pork loin	He or mixtures He+O_2_; 10 slpm; 3 kV; 30 kHz; 3 mm distance	*Listeria monocytogenes* ~0.59 log *Escherichia coli* ~0.55 log, in 10 min	Loss of lightness. Significant reductions in sensory quality parameters (appearance, color, odor, acceptability).	Kim et al., 2013
Pork	Mixtures of N_2_ and O_2_; 15 kHz; 2 W	*Listeria monocytogenes* ~2.0 log *Escherichia coli* O157:H7 ~2.5 log *Salmonella Typhimurium* ~2.7 log, in 10 min	Minor changes in color and taste. No effects in texture.	Jayasena et al., 2015
Frozen and unfrozen pork	Air; 2.5 m/s; 20 kV; 58 kHz; 25 mm distance	*Listeria monocytogenes* ~1.0 log *Escherichia coli* O157:H7 ~1.5 log, in 2 min	Only significant impact on the sensory characteristics (color and appearance) of unfrozen pork	Choi et al., 2016
Beef	N_2_+O_2_; 15 kHz; 2 W	*Listeria monocytogenes* ~1.9 log *Escherichia coli* O157:H7 ~2.6 log *Salmonella Typhimurium* ~2.6 log, in 10 min	Minor changes in color and taste. No effects in texture.	Jayasena et al., 2015
Chicken meat and skin	He (5 L/min)+O_2_ (100 mL/min); 16 kV; 30 kHz; 1 cm distance	*Listeria innocua* ~1.0 log on skin, in 8 min >3.0 log on meat, in 4 min	Not assessed	Noriega et al., 2011
Chicken breast and thigh	Air	*Salmonella enterica* ~2.5 log *Campylobacter jejuni* ~2.4 log, in 3 min	Not assessed	Dirks et al., 2012
Beef jerky	Ar; 20,000 sccm; 200 W; 1 cm distance	*Staphycococcus aureus* ~1.8 log, in 8 min	No changes in texture and color	Kim et al., 2014
	Air; 15 kHz	*Listeria monocytogenes*, ~2.4 log *Escherichia coli* O157:H7, ~2.7 log *Salmonella* Typhimurium, ~3.0 log *Aspergillus flavus*, ~3.2 log, in 10 min	No significant changes in metmyoglobin content, shear force, and myofibrillar fragmentation index; changes in peroxides content and color parameters; negative effects on flavor, off-odor, and overall acceptability	Yong et al., 2017
Cooked chicken breast	N_2_ (7 L/min)+O_2_ (0.07 L/min); 2 kV; 50 kHz; 4 cm distance	*Listeria monocytogenes* ~4.7 log, in 2 min	Not assessed	Lee et al., 2011
Cooked ham	N_2_ (7 L/min)+O_2_ (0.07 L/min); 2 kV; 50 kHz; 4 com distance	*Listeria monocytogenes* ~6.5 log, in 2 min	Not assessed	Lee et al., 2011
Ham	He; 13.56 MHz; 150 W; 0,6 mm distance	*Listeria monocytogenes* ~1.7 log, in 2 min	Not assessed	Song et al., 2009
Bacon	He (10 L/min)+O_2_ (10 sccm); 13,56 MHz; 125 W; 3 mm distance	*Listeria monocytogenes* ~2.6 log *Escherichia coli* ~3.0 log *Salmonella* Typhimurium ~1.0 log, Total aerobic bacteria ~4.6 log, in 1.5 min	No physical damage on surface tissues. Higher lightness. Lipid oxidation was not observed	Kim et al., 2011
Bresaola	Ar (70%) + O_2_ (30%); 27.8 kV; 15.5 W	*Listeria innocua* >1.5 log, in 10 s	Loss of redness, Induced lipid oxidation.	Rød et al., 2012
**DAIRY PRODUCTS**				
Cheese	He; 13.56 MHz; 150 W; 0,6 mm distance	*Listeria monocytogenes* ~8.5 log, in 2 min	Not assessed	Song et al., 2009
	Air; 15 kHz; 250 W	*Escherichia coli* ~2.7 log, in 1 min *Salmonella* Typhimurium ~3.1 log, in 45 s	Not assessed	Yong et al., 2015a
Cheddar cheese	Air; 15 kHz; 2 W	*Listeria monocytogenes* ~5.8 log *Escherichia coli* O157:H7 ~3.6 log *Salmonella* Typhimurium ~2.1 log, in 10 min	No changes in color and sensory appearance. Significant reductions in flavor and overall acceptance. Increased off-odor	Yong et al., 2015b
**EGGS**				
Cooked egg white	N_2_ (7 L/min)+O_2_ (0.07 L/min); 2 kV; 50 kHz; 4 cm distance	*Listeria monocytogenes* ~6.7 log Total aerobic bacteria ~2.9 log, in 2 min	Loss of lightness. No impact on sensory attributes (color, flavor, texture, taste, acceptability).	Lee et al., 2012b
Cooked egg yolk	N_2_ (7 L/min)+O_2_ (0.07 L/min); 2 kV; 50 kHz; 4 cm distance	*Listeria monocytogenes* ~7.1 log Total aerobic bacteria ~2.3 log, in 2 min	Loss of lightness. Significant reductions in flavor, taste and overall acceptability	Lee et al., 2012b
**FISH AND SEAFOOD**				
Dried squid shreds	Air; 2 L/s; 20 kV; 58 kHz; 25 mm distance	*Staphylococcus aureus* ~0.9 log Marine bacteria ~1.6 log Aerobic bacteria ~2.0 log Yeast and molds ~0.9 log, in 3 min	No significant changes in color characteristics and volatile basic nitrogen content; moisture and thiobarbituric acid reactive substances levels were altered; no significant impact on sensory characteristics (color, flavor, taste, texture, acceptability, overall acceptance)	Choi et al., 2017
**FRUIT, CEREALS AND VEGETABLES**				
Blueberries	Air; 4 cfm; 47 KHz; 549 W; 7.5 cm distance	Total aerobic count ~0.8 log Yeast and molds ~1.2 log, in 2 min	Significant reductions in firmness and anthocyanins; surface color was significantly impacted	Lacombe et al., 2015
Strawberries	Air; 70 kV; 50 Hz; 140–160 mm distance	*Listeria monocytogenes* ~4.2 log *Escherichia coli* O157:H7 ~3.5 log *Salmonella* Typhimurium ~3.8 log, in 5 min	Not assessed	Ziuzina et al., 2014
Pears	Air; 5 slm; 500 V	*Salmonella* spp. ~0.2 log, in 0.5 s	Changes in physiochemical properties were within an acceptable range	Wang et al., 2012
Apples	Ar (5 L/min)+O_2_ (0.1%); 10 kV; 8 W; 17 mm distance	*Escherichia coli* ~4.7 log, in 1 min	Not assessed	Baier et al., 2014
Cherry tomatoes	Air; 70 kV; 50 Hz; 140–160 mm distance	*Listeria monocytogenes* ~6.7 log, in 2 min *Escherichia coli* ~3.1 log, in 1 min *Salmonella* Typhimurium ~6.3 log, in 10 s Aerobic mesophilic bacteria ~4.0 log, in 2 min Yeast and molds ~5.0 log, in 2 min	Not assessed	Ziuzina et al., 2014
Tomato	Ar (5 L/min)+O_2_ (0.1%); 10 kV; 8 W; 17 mm distance	*Escherichia coli* ~3.3 log, in 1 min	Not assessed	Baier et al., 2014
Corn salad	Ar (5 L/min)+O_2_ (0.1%); 10 kV; 8 W; 17 mm distance	*Escherichia coli* ~4.1 log, in 1 min	No changes in color were observed	Baier et al., 2014
Cucumber	Air; 5 slm; 500 V	*Salmonella* spp. ~0.4 log, in 0.5 s	Changes in physiochemical properties were within an acceptable range	Wang et al., 2012
	Ar (5 L/min)+O_2_ (0.1%); 10 kV; 8 W; 17 mm distance	*Escherichia coli* ~4.7 log, in 1 min	Not assessed	Baier et al., 2014
Carrots	Air; 5 slm; 500 V	Salmonella spp. ~1.0 log, in 0.5 s	Changes in physiochemical properties were within an acceptable range	Wang et al., 2012
	Air (20 L/min); 2.45 Ghz; 1.2 kW	*Escherichia coli* ~4.5 log Mesophilic aerobic counts ~3.5 log, in 2 min	Significant effects on color. No impact on elastic properties	Baier et al., 2015
Lettuce	Air; 4.5 com distance	*Aeromonas hydrophila* ~5 log, in 15 s	Not assessed	Jahid et al., 2014
Rapeseed seeds	Air (2 L/min); 20 kV; 58 kHz; 25 mm distance	*Escherichia coli* ~2.0 log *Bacillus cereus* ~1.2 log *Salmonella spp*. ~1.8 log Total aerobic bacteria ~2.2 log Yeast and molds ~2.0 log, in 3 min	Physicochemical (weight, length, moisture content) and sensory characteristics (appearance, color, flavor, taste, texture) were unaffected.	Puligundla et al., 2017
Orange juice	Air; 20 kV; 60 kHz; 1.14 W/cm^2^; batch	*Escherichia coli* ~5.0 log, in 8 s *Staphylococcus aureus* ~5.0 log, in 12 s *Candida albicans* ~5.0 log, in 25 s	Almost no effect on vitamin C content, pH, turbidity or °Brix	Shi et al., 2011
Black peppercorns	Air (20 L/min) +Ar (14 L/min); 4 cm distance	*Salmonella* ~5.0 log, in 80 sec	Minimal changes in color	Sun et al., 2014
Almonds	Air; 30 kV; 2 kHz	*Escherichia coli* ~5 log, in 30 sec	Not assessed	Deng et al., 2007
	Air; 47 kHz; 549 W; 6 cm distance	*Escherichia coli* O157:H7 ~1.34 log, in 20 s	No gross changes in color, aroma and surface features	Niemira, 2012
Wheat grains	Ar (28 L/min); 8 kV; 10 kHz	*Geobacillus stearothermophillus* spores ~3.0 log, in 60 min	Functional wheat grain properties (falling number, gluten content) were not negatively affected.	Butscher et al., 2016

*slpm, standard liters per minute; sccm, standard cubic centimeters per minute; cfm, cubit feet meter*.

Overall, there is a unanimous agreement in that on smooth and polished surfaces the anti-microbial efficiency of NTAP is very high (Noriega et al., 2011; Bermúdez-Aguirre et al., 2013; Fernández et al., 2013; Kim et al., 2014; Butscher et al., 2016; Cui et al., 2016a), whereas rough, porous and irregular surfaces, such as those of some foods, offer numerous places for microorganisms to fix and hide, thus avoiding the action of plasma (Fernández et al., 2012; Bermúdez-Aguirre et al., 2013; Butscher et al., 2016; Cui et al., 2016a). In fact, some studies using electron microscopy have shown that inoculated microorganisms are capable of finding shelter within various food irregularities, such as cracks, grooves or gaps (Fernández et al., 2013; Jahid et al., 2014; Ziuzina et al., 2014; Cui et al., 2016a).

## Applications of NTAP in the Food Industry

Once the mechanisms of microbial inactivation by NTAP and the factors that determine its lethal efficacy have been described, the following section of the review article will discuss the potential of this novel technology for specific applications within food processing industries.

### NTAP as a Decontamination Technique

NTAP can be used as a decontamination technique for foods, packaging materials, equipment, and even the processing environment itself.

In relation to surface decontamination of foods, several studies have shown the potential of NTAP to improve the microbiological quality of a wide range of solid foods, including strawberries, tomatoes, chicken breast filets, ham, cheese slices, carrots, melon, or lettuce, and liquid foods, such as milk, apple and orange juices, and coconut liquid endosperm (Table 2). Although microbial inactivation rates obtained widely vary among studies, surfaces and microbial species tested, promising results have been reported. For example, with 2 min treatments, 4 to 8 logarithmic reductions were obtained for *L. monocytogenes* in tomatoes, cooked chicken breast filets and ham, cooked egg white and egg yolk, and cheese slices (Song et al., 2009; Lee et al., 2011, 2012b; Ziuzina et al., 2014), and 4.5 log units for *E. coli* in carrots (Baier et al., 2015a). Even treatments as short as 10 and 15 s have been able to reduce the population of *A. hydrophila* in lettuce in 5 log cycles (Jahid et al., 2014) and that of *S*. Typhimurium in tomatoes in 6 log units (Ziuzina et al., 2014). However, most studies have not evaluated the impact of NTAP treatments on the nutritional and sensory properties of such foods, despite the fact that plasma-generated reactive species could lead to organoleptic changes in the end product. Moreover, information available in the literature is very variable, with some authors identifying minor changes in color, texture, appearance, taste or aroma of foods, while others have reported important modifications in sensory attributes after NTAP treatment (Table 2). The observed discrepancies are probably due to the variability in equipment, plasma generation conditions or type of food used in validation studies. Indeed, several authors have shown how these factors influence the maintenance of food sensory attributes. For example, the application of similar NTAP treatments significantly modified the color of carrots (Baier et al., 2015) and the taste of cooked egg yolk (Lee et al., 2012b), but did not affect these quality attributes in apples and cooked egg white, respectively. Moreover, NTAP treatments using helium as working gas caused changes in pork loin color, whereas this quality attribute was not modified in treatments using a mixture of helium and oxygen (Kim et al., 2013). Baier et al. (2014) also reported changes in leaf lettuce color after a NTAP treatment, which only occurred when the distance between food and plasma generation source was low (5 mm). Furthermore, Rød et al. (2012) described that an increase in potency, treatment time and storage time was accompanied by an increased lipid oxidation and rancidity of bresaola. Similarly, a NTAP treatment of 5–10 min has been shown to induce lipid oxidation in cheese samples (Yong et al., 2015b). However, no significant changes in the fatty acid composition of beef jerky were detected after exposure to plasma for 5 min (Kim et al., 2014).

Although the vast majority of studies have focused on evaluating the potential of NTAP for the decontamination of solid foods, some authors have addressed its efficacy in liquid foods, obtaining also in some occasions promising results. Thus, up to 5 log reductions have been obtained for *E. coli* in orange and apple juice after a treatment of 8 and 40 s, respectively, and treatments of 12 and 25 s had a similar lethality effect for *S. aureus* and *Candida albicans*, respectively (Shi et al., 2011; Liao et al., 2018).

NTAP can be also used for the decontamination of food packaging materials. Currently, the most widely used methods with this aim are based on the employment of dry or wet heat or chemical agents, such as peracetic acid or hydrogen peroxide, which can be applied at relatively high temperatures, ranging from 65 to 80°C, to increase their effectiveness. However, most polymers used as packaging materials do not withstand high temperatures. In addition, some chemical agents also present other drawbacks, such as their difficult handling, maximum limits allowed and the possible generation of toxic waste substances. NTAP is an attractive alternative to chemical decontamination agents, due to its effectiveness as a decontamination technique which successfully inactivates microorganisms, including bacterial and fungal spores, on abiotic surfaces (Muranyi et al., 2007, 2008, 2010; Klämpfl et al., 2012). For example, Muranyi et al. (2007), assessing the efficacy of NTAP for the inactivation of vegetative cells (*E. coli, S. aureus, S*. Mons, and *D. radiodurans*), bacterial spores (*B. atrophaeus, B. pumilus, C. botulinum, C. sporogenes*) and fungi spores (*A. niger*) on PET, achieved, with treatment times as short as 1 second, between 5.6 and 6.9 log reductions for vegetative cells, between 5.1 and 6.1 log reductions for bacterial spores and 3 log reductions for *A. niger* conidiospores.

NTAP can even be used for the decontamination of food once packaged, using dielectric barrier discharge equipment (Fröhling et al., 2012a; Rød et al., 2012; Ziuzina et al., 2014; Jayasena et al., 2015). The reactive species generated in this case would simultaneously decontaminate both the packaging material and the food itself. Moreover, once the treatment concludes, the plasma species recombine, the atmosphere returns to its initial composition, and recontamination of the end product after NTAP treatment is thus avoided. The use of NTAP for the decontamination of food once packaged, has been successfully studied in cherry tomatoes, strawberries, bresaola and pork and beef meat by several research groups (Fröhling et al., 2012a; Rød et al., 2012; Ziuzina et al., 2014; Jayasena et al., 2015).

Another potential application of NTAP in the food industry is the decontamination of food processing surfaces and equipment. In fact, a novel plasma system has been designed to decontaminate slicers, in which the cutting blade of the equipment constitutes one of the electrodes of the dielectric discharge system (Leipold et al., 2010). These authors obtained an effective inactivation of *L. innocua* inoculated on the cutting blade, representing a novel approach for decontaminating food processing equipment which could be applied to other surfaces, such as conveyors.

Recently, several authors have described the fact that water treated by NTAP, so-called plasma-activated water (PAW), has relevant antimicrobial activity, which persist over a long period of time (Kamgang-Youbi et al., 2009; Ercan et al., 2013; Zhang et al., 2013). This offers new possibilities for decontamination of both surfaces and food, through the treatment of water by NTAP, which will be then used for decontamination purposes. This approach may be a promising alternative to the chemical agents currently used for the sanitation of surfaces, equipment and minimally processed vegetables. In fact, the potential of plasma-activated water has recently been revealed for the decontamination of strawberries (Ma et al., 2015) and mushrooms (Xu et al., 2016). Indeed, a bactericidal effect similar to that obtained using sodium hypochlorite solutions (Issa-Zacharia et al., 2010), with no changes in color or firmness, was observed for strawberries treated with plasma-activated water, with log reductions for *S. aureus* ranging from 1.7 to 2.3 log depending on the exposure time (Ma et al., 2015). Log reductions achieved following a similar approach for treated mushrooms were between 1.5 and 0.7 log after a week of storage, and mushrooms maintained their initial quality attributes (Xu et al., 2016).

### Other Applications of NTAP in Food Processing

Apart from being used as a decontamination technique, NTAP may also represent an alternative food processing method which can be used, for example, to obtain the characteristic color of cured meat products without adding nitrites, extend the shelf-life of oils used for coating cookies, reduce the cooking time of cereals, improve the extraction of essential oils or modify the functional properties of flours.

As already mentioned, the interaction of plasma with water results in the generation of reactive oxygen and nitrogen species, including nitrates and nitrites (Oehmigen et al., 2010), reaching concentrations of up to 1,050 ppm (Hao et al., 2014). The generation of nitrites during plasma treatment may raise a flag since use of nitrites in food is restricted, with maximum allowed concentrations, due to their potential toxicological effects. However, Jung et al. (2015) manufactured frankfurters by replacing the nitrites of the curing salts by plasma-treated water and did not detect significant differences in their microbiological or organoleptic quality during 28 days of storage at refrigeration. Interestingly, the residual nitrite content was 30% lower in sausages processed with plasma-treated water. Similar results have been also obtained by directly treating the meat butter with NTAP at different stages of the sausage making process, and a patent for NTAP as a system to eliminate nitrites in meat products has been recently filed (Lim et al., 2015).

NTAP can be used to modify the surface properties of various materials, such as paper, polymers or electronic equipment, to confer them certain functional properties of interest. The idea of using this technology to achieve surface modifications in foods is innovative. NTAP has been used to modulate hydrophobicity or hydrophilicity of oil coatings on cookies, in order to improve palatability and appearance (Misra et al., 2014). These authors demonstrated that NTAP increased surface hydrophobicity, resulting in a greater extension of the incorporated oil and in a more accurate infiltration of oil, without affecting the color, odor and appearance of cookies. Thus, it allowed the preservation of the functionality of the sprayed oil, also decreasing the amount of oil required, which is interesting from a nutritional and economic point of view. A modification of surface properties after NTAP treatments has also been observed for basmati rice (Thirumdas et al., 2015). In this case, NTAP increased the hydrophilicity and water absorption rate of the cereal, and decreased the cooking time, from 20 to 13 min. Electron microscopy images of the treated rice grains revealed the presence of cracks and depressions on their surface, which probably provide routes for faster absorption of water. Although this experiment was carried out under vacuum conditions, which does not allow a direct extrapolation of the results, similar results are expected at atmospheric pressure conditions. Similarly, NTAP is currently being also proposed as a promising technology for the physico-chemical modification of processing surfaces, such as those of food-contact materials, in a way to prevent microbial adhesion and formation of biofilms, therefore limiting episodes of food cross-contamination with persistent microorganisms colonizing food processing environments. These surface modification techniques are sometimes focused at changing electronegativity, hydrophobicity or morphology/topography of surfaces (Bazaka et al., 2015).

Another possible application of NTAP is its use to improve those processes that involve mass transfer. Indeed, exposure of lemon skins to plasma has been shown to increase the yield of extracted essential oils (Kodama et al., 2014). However, these results should be taken with caution, since essential oils can be oxidized to a certain extent, depending on the gas used for NTAP generation.

Finally, NTAP has been also evaluated as a methodology capable of inducing modifications in the functional properties of proteins. Misra et al. (2015) showed that the secondary structure of gluten became more stable when wheat flour had been treated with an air plasma, and observed significant changes in the rheological properties of the doughs obtained. These changes, both in viscosity and elasticity, depended on the treatment conditions, applied voltage and exposure time. Thus, NTAP can be used as an innovative strategy in order to modulate the functionality of wheat flour during processing of bread, pasta, noodles, cookies, and others.

## Conclusions and Future Prospects

NTAP is a promising food decontamination technology capable of inactivating bacteria, yeasts, molds, fungal and bacterial spores both on abiotic surfaces (e.g., packaging materials, food processing environments and equipment) and on foods. In addition, NTAP has also other different innovative applications of great interest for food quality and safety improvement.

Although the exact mechanism of microbial inactivation by NTAP is not completely known yet, the cellular envelopes, DNA and proteins are recognized as potential targets, and damages produced to them are the result of the simultaneous action of the different components and reactive species occurring in plasma when the microbial detoxifying capacity is overcome, resulting in the accumulation of dysfunctional macromolecules that compromise cellular viability. The multi-target nature of NTAP could justify the great antimicrobial effectiveness of NTAP in comparison with other technologies that affect a single component or cellular structure. The relative contribution of each individual damage to the total lethal effect of NTAP is unknown yet, since, on the one hand, the composition of plasmas may be very different depending on the conditions of production and, on the other hand, the magnitude of the damage could be in turn dependent on the type of microorganism and the time of treatment (Muranyi et al., 2010; Fröhling et al., 2012b; Tseng et al., 2012; Han et al., 2013, 2016).

Inadequate reporting of experimental methodologies may hinder independent verification and validation of results among different laboratories and equipment units. It is therefore advisable to establish a set of recommended guidelines for conducting and reporting NTAP experiments which would help improve the reliability of future experimental data, thus facilitating process optimization for industrial implementation. Some suggestions on this regard are provided in Table 3.

**Table 3:** Suggested information to be provided in research studies related to non-thermal atmospheric plasma.

**Category**	**Variable and minimum description to be included**
Process equipment	Type of equipment, type of discharge, and electrode configuration
	Equipment model and vendor name
	Distance between the point of plasma generation and the sample
Processing conditions	Type of gas
	Gas flow rate and gas moisture content
	Energy supplied: voltage, power, and frequency
	Processing time
	Composition of the generated plasma
Food or sample	Type of food or sample: detailed description
	Water activity, pH and composition of the sample
	Time—temperature history
Microbial factors	Genus, species, and strain(s) used
	Initial microbial load
	Pre-history of the microbial inoculum
	Description of the procedure followed for preparing the inoculum and enumerating microorganisms

However, the industrial implementation of NTAP with these aims still requires substantial research efforts, which should be especially focused at (i) assessing the impact that NTAP shows on the nutritional and sensory quality of treated foods in order to facilitate the design of preservation regimes capable of inhibiting pathogenic and spoilage microorganisms while maintaining food quality attributes; (ii) identifying the plasma components and reactive species responsible for the antimicrobial activity of NTAP, which will allow a better selection of processing conditions; (iii) confirming the lack of toxicity of the chemical species generated during NTAP treatments; (iv) developing combined preservation treatments, within a hurdles technology concept, where other inactivation approaches showing a synergic effect when applied together with NTAP will be used; (v) and designing fit-for-purpose equipment susceptible to be easily adopted in a processing line, being compact, energetically efficient, and cost-effective.

## Author Contributions

All authors listed have made a substantial, direct and intellectual contribution to the work, and approved it for publication.

### Conflict of Interest Statement

The authors declare that the research was conducted in the absence of any commercial or financial relationships that could be construed as a potential conflict of interest.
